# Poly[[dodeca­aqua­hexa­kis­(μ_2_-pyridine-2,5-dicarboxyl­ato)tricopper(II)diytterbium(III)] dihydrate]

**DOI:** 10.1107/S1600536811007446

**Published:** 2011-03-09

**Authors:** Fwu Ming Shen, Shie Fu Lush

**Affiliations:** a Department of Biotechnology, Yuanpei University, HsinChu 30015, Taiwan; b Department of General Education Center, Yuanpei University, HsinChu 30015, Taiwan

## Abstract

The asymmetric unit of the title heterometallic coordination polymer, {[Cu_3_Yb_2_(C_7_H_3_NO_4_)_6_(H_2_O)_12_]·2H_2_O}_*n*_, contains one Yb^III^ and two Cu^II^ atoms. The Cu^II^ atom that is located on an inversion center is *N*,*O*-chelated by two pyridine-2,5-dicarboxyl­ate (pdc) anions in a square-planar geometry; the Cu atom located on a general position is *N*,*O*-chelated by two pdc anions in the basal plane and is further coordinated by a water O atom at the apical position in a distorted square-pyramidal geometry. The Yb(III) atom is eight coordinated in a distorted square-anti­prismatic geometry formed by three carboxyl­ate O atoms from three pdc anions and five water mol­ecules. The pdc anions bridge adjacent Yb(III) and Cu(III) atoms, forming a three-dimensional polymeric structure. The crystal structure contains extensive O—H⋯O hydrogen bonds. π–π stacking is present in the crystal structure, the shortest centroid–centroid distance between parallel pyridine rings of adjacent mol­ecules being 3.646 (3) Å.

## Related literature

For general background to the use of pdc as a ligand in rare earth transition metal complexes, see: Huang *et al.* (2008 ▶). For related structures, see: Wei *et al.* (2005 ▶); Wen *et al.* (2007 ▶).
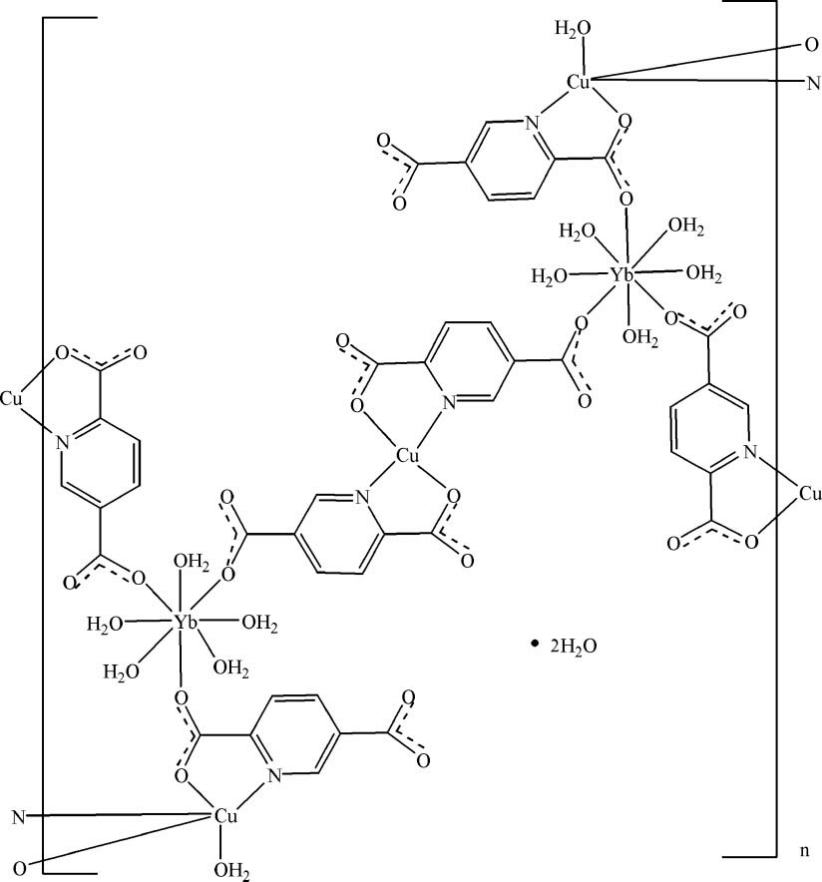


## Experimental

### 

#### Crystal data

[Cu_3_Yb_2_(C_7_H_3_NO_4_)_6_(H_2_O)_12_]·2H_2_O
*M* *_r_* = 1815.58
Triclinic, 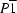

*a* = 7.3486 (4) Å
*b* = 13.5417 (7) Å
*c* = 15.1244 (8) Å
α = 72.534 (1)°
β = 76.330 (1)°
γ = 80.166 (1)°
*V* = 1386.89 (13) Å^3^
*Z* = 1
Mo *K*α radiation
μ = 4.59 mm^−1^
*T* = 295 K
0.13 × 0.08 × 0.05 mm

#### Data collection

Bruker SMART 1000 CCD area-detector diffractometer
Absorption correction: multi-scan (*SADABS*; Bruker, 2001 ▶) *T*_min_ = 0.739, *T*_max_ = 0.973
14910 measured reflections
6624 independent reflections
6010 reflections with *I* > 2σ(*I*)
*R*_int_ = 0.044

#### Refinement

*R*[*F* ^2^ > 2σ(*F* ^2^)] = 0.049
*wR*(*F* ^2^) = 0.083
*S* = 1.23
6624 reflections
421 parameters
H-atom parameters constrained
Δρ_max_ = 1.01 e Å^−3^
Δρ_min_ = −1.68 e Å^−3^

### 

Data collection: *SMART* (Bruker, 2007 ▶); cell refinement: *SAINT* (Bruker, 2007 ▶); data reduction: *SAINT*; program(s) used to solve structure: *SHELXTL* (Sheldrick, 2008 ▶); program(s) used to refine structure: *SHELXTL* (Sheldrick, 2008 ▶); molecular graphics: *PLATON* (Spek, 2009 ▶); software used to prepare material for publication: *PLATON*.

## Supplementary Material

Crystal structure: contains datablocks I, global. DOI: 10.1107/S1600536811007446/xu5166sup1.cif

Structure factors: contains datablocks I. DOI: 10.1107/S1600536811007446/xu5166Isup2.hkl

Additional supplementary materials:  crystallographic information; 3D view; checkCIF report

## Figures and Tables

**Table 1:** Selected bond lengths (Å)

Yb1—O2	2.299 (5)
Yb1—O3	2.272 (4)
Yb1—O4	2.411 (4)
Yb1—O5	2.278 (4)
Yb1—O6	2.364 (4)
Yb1—O7	2.447 (3)
Yb1—O13	2.364 (4)
Yb1—O15	2.281 (4)
Cu1—N1	1.975 (4)
Cu1—N2^i^	1.955 (4)
Cu1—O1	2.372 (4)
Cu1—O8	1.964 (3)
Cu1—O11^i^	1.943 (3)
Cu2—N3	1.967 (4)
Cu2—O17	1.931 (4)

Symmetry code: (i) 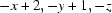.

**Table 2:** Hydrogen-bond geometry (Å, °)

*D*—H⋯*A*	*D*—H	H⋯*A*	*D*⋯*A*	*D*—H⋯*A*
O1—H1*A*⋯O17^ii^	0.82	2.50	3.072 (5)	128
O1—H1*A*⋯O18^ii^	0.82	2.06	2.865 (6)	166
O1—H1*B*⋯O9^iii^	0.82	2.02	2.840 (6)	173
O2—H2*A*⋯O10^iv^	0.82	2.48	2.988 (7)	121
O2—H2*B*⋯O19	0.82	1.83	2.644 (8)	173
O3—H3*A*⋯O16	0.82	1.91	2.572 (6)	137
O3—H3*B*⋯O10^iii^	0.82	1.91	2.695 (6)	159
O4—H4*A*⋯O9^iii^	0.82	1.87	2.670 (6)	165
O4—H4*B*⋯O14^v^	0.82	1.98	2.774 (6)	163
O5—H5*A*⋯O14	0.82	1.85	2.586 (6)	148
O5—H5*B*⋯O8	0.82	2.06	2.717 (6)	137
O6—H6*A*⋯O1^vi^	0.82	2.09	2.826 (6)	149
O6—H6*B*⋯O4^vi^	0.82	2.15	2.936 (6)	160
O19—H19*A*⋯O20^vii^	0.82	2.06	2.816 (8)	152
O19—H19*B*⋯O12^viii^	0.82	2.15	2.965 (7)	171
O20—H20*A*⋯O13^v^	0.82	2.55	3.174 (7)	134
O20—H20*B*⋯O16	0.82	2.15	2.957 (9)	166

Symmetry codes: (ii) 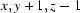; (iii) 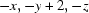; (iv) 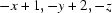; (v) 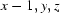; (vi) 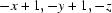; (vii) 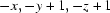; (viii) 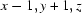.

**Fig. 1.**
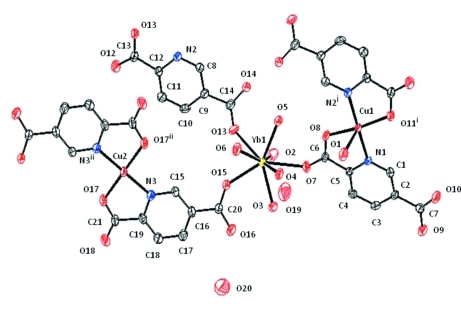
View of the title compound. Displacement ellipsoids for non-H atoms are drawn at the 50% probability level. H atoms have been omitted for clarity.[Symmetry codes: (i) 2 - x, 1 - y, -z; (ii)1 - x, -y, 1 - z].

**Fig. 2.**
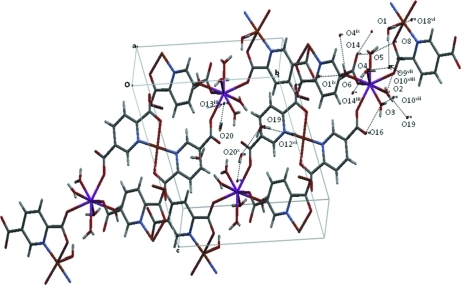
The molecular packing for the title compound. Hydrogen bonds are shown as dashed lines.

## supplementary crystallographic information

### Comment

Many studies concering the use of pdc as a ligand toward transition metal (Wen *et al.*, 2007) and /or rare earth transition metal (Wei *et al.*, 2005; Huang *et al.*, 2008) have shown that a great variety of polymeric structures can be obtained as a result of the differnet coordination modes of the pdc ligands.

The asymmetric unit of the title heterometallic coordination polymer, [Cu_3_Yb_2_(C_7_H_3_NO_4_)_6_(H_2_O)_12_.4H_2_O]_n_,contains one Yb^III^ and two Cu^II^ atoms, three pyridine-2,5-dicarboxylate (pdc) anions and six water molecules. One Cu^II^ atom is located on an located on an inversion center and is N,O-chelated by two pdc anions in the equatorial plane with square-planar geometry; the other Cu atom is N,O-chelated by two pdc anions in the coordination basal plane and coordinated by a carboxyl O atom at the apical position with a distorted square-pyramidal geometry [Cu—O = 2.374 (4) Å in the apical direction]. The Yb atom is eight coordinated with a distorted square-antiprismatic geometry formed by three carboxylate O atoms from three pdc anions and five water molecules (selected bond lengths are given in Table 1). The pdy anions bridge adjacent Yb and Cu atoms to form the three dimensional polymeric structure (Fig. 1).

The crystal structure contains the extensive O—H···O and weak C—H···O hydrogen bonds (Fig. 2 and Table 2). π-π stackings are present in the crystal structure, the shortest centroids distance between parallel pyridine rings is 3.646 (3) Å [Cg6^iiv^···Cg6 (N1/C1—C5)] [symmetry code: (iiv) -*x*, 2 - *y*, -*z*].

### Experimental

A mixture of ytterbium chloride hexahydrate (0.2438 g, 0.25 mmol), copper acetate hydrate (0.050 g, 0.25 mmol), pyridine-2,5-dicarboxylic acid (0.0418 g, 0.25 mmol,) and 10 ml H_2_O were put in a 23-ml Teflon liner reactor and heated at 418 K in oven for 48 h. The resulting solution was slowly cooled to room temperature. The blue transparent single crystals of the title complex were obtained in 34.26% yield (based on Yb).

### Refinement

Water H atoms were placed in calculated positions and refined with the distances constrains of O—H = 0.82, and *U*_iso_(H)= 1.5*U*_eq_(O). Other H atoms were positioned geometrically with C—H = 0.93 Å and refined using a riding model, *U*_iso_(H) = 1.2*U*_eq_(C).

### Crystal data

**Table:**

[Cu_3_Yb_2_(C_7_H_3_NO_4_)_6_(H_2_O)_12_]·2H_2_O	*Z* = 1
*M**_r_* = 1815.58	*F*(000) = 891
Triclinic, *P*1	*D*_x_ = 2.174 Mg m^−^^3^
Hall symbol: -P 1	Mo *K*α radiation, λ = 0.71073 Å
*a* = 7.3486 (4) Å	Cell parameters from 7576 reflections
*b* = 13.5417 (7) Å	θ = 2.5–25.0°
*c* = 15.1244 (8) Å	µ = 4.59 mm^−^^1^
α = 72.534 (1)°	*T* = 295 K
β = 76.330 (1)°	Columnar, blue
γ = 80.166 (1)°	0.13 × 0.08 × 0.05 mm
*V* = 1386.89 (13) Å^3^	

### Data collection

**Table:**

Bruker SMART 1000 CCD area-detector diffractometer	6624 independent reflections
Radiation source: fine-focus sealed tube	6010 reflections with *I* > 2σ(*I*)
graphite	*R*_int_ = 0.044
Detector resolution: 9 pixels mm^-1^	θ_max_ = 28.3°, θ_min_ = 1.4°
φ and ω scans	*h* = −9→9
Absorption correction: multi-scan (*SADABS*; Bruker, 2001)	*k* = −18→18
*T*_min_ = 0.739, *T*_max_ = 0.973	*l* = −20→20
14910 measured reflections	

### Refinement

**Table:**

Refinement on *F*^2^	Primary atom site location: structure-invariant direct methods
Least-squares matrix: full	Secondary atom site location: difference Fourier map
*R*[*F*^2^ > 2σ(*F*^2^)] = 0.049	Hydrogen site location: inferred from neighbouring sites
*wR*(*F*^2^) = 0.083	H-atom parameters constrained
*S* = 1.23	*w* = 1/[σ^2^(*F*_o_^2^) + (0.019*P*)^2^ + 3.1503*P*] where *P* = (*F*_o_^2^ + 2*F*_c_^2^)/3
6624 reflections	(Δ/σ)_max_ = 0.001
421 parameters	Δρ_max_ = 1.01 e Å^−^^3^
0 restraints	Δρ_min_ = −1.67 e Å^−^^3^

### Special details

**Table:**

Geometry. All e.s.d.'s (except the e.s.d. in the dihedral angle between two l.s. planes) are estimated using the full covariance matrix. The cell e.s.d.'s are taken into account individually in the estimation of e.s.d.'s in distances, angles and torsion angles; correlations between e.s.d.'s in cell parameters are only used when they are defined by crystal symmetry. An approximate (isotropic) treatment of cell e.s.d.'s is used for estimating e.s.d.'s involving l.s. planes.
Refinement. Refinement of *F*^2^ against ALL reflections. The weighted *R*-factor *wR* and goodness of fit *S* are based on *F*^2^, conventional *R*-factors *R* are based on *F*, with *F* set to zero for negative *F*^2^. The threshold expression of *F*^2^ > σ(*F*^2^) is used only for calculating *R*-factors(gt) *etc*. and is not relevant to the choice of reflections for refinement. *R*-factors based on *F*^2^ are statistically about twice as large as those based on *F*, and *R*- factors based on ALL data will be even larger.

### Fractional atomic coordinates and isotropic or equivalent isotropic displacement parameters (Å^2^)

**Table:**

	*x*	*y*	*z*	*U*_iso_*/*U*_eq_	
Yb1	0.46140 (3)	0.553608 (18)	0.188850 (15)	0.01980 (7)	
Cu1	0.55405 (9)	0.88959 (5)	−0.15334 (4)	0.02228 (15)	
Cu2	0.5000	0.0000	0.5000	0.0251 (2)	
O1	0.3238 (6)	0.7999 (3)	−0.1743 (3)	0.0346 (10)	
H1A	0.2626	0.8487	−0.2052	0.052*	
H1B	0.2574	0.7825	−0.1215	0.052*	
O2	0.5782 (7)	0.6367 (4)	0.2722 (3)	0.0489 (12)	
H2A	0.6913	0.6273	0.2718	0.073*	
H2B	0.5274	0.6672	0.3126	0.073*	
O3	0.1694 (6)	0.6205 (3)	0.2490 (3)	0.0416 (12)	
H3A	0.1014	0.6001	0.3008	0.062*	
H3B	0.1087	0.6689	0.2174	0.062*	
O4	0.2936 (5)	0.5798 (3)	0.0631 (3)	0.0280 (9)	
H4A	0.2306	0.6367	0.0528	0.042*	
H4B	0.2172	0.5369	0.0803	0.042*	
O5	0.6992 (5)	0.5902 (3)	0.0603 (3)	0.0322 (10)	
H5A	0.8033	0.5564	0.0583	0.048*	
H5B	0.7171	0.6486	0.0256	0.048*	
O6	0.4981 (6)	0.4061 (3)	0.1308 (3)	0.0329 (9)	
H6A	0.5319	0.3489	0.1639	0.049*	
H6B	0.5320	0.4054	0.0754	0.049*	
O7	0.4094 (5)	0.7414 (3)	0.1179 (2)	0.0276 (9)	
O8	0.5572 (5)	0.7817 (3)	−0.0331 (2)	0.0249 (8)	
O9	−0.0731 (6)	1.2456 (3)	−0.0036 (3)	0.0334 (10)	
O10	0.0874 (6)	1.2581 (3)	−0.1509 (3)	0.0387 (11)	
O15	0.3506 (6)	0.4262 (3)	0.3191 (3)	0.0395 (11)	
O16	0.0621 (7)	0.4704 (4)	0.3939 (3)	0.0575 (15)	
O19	0.3979 (9)	0.7458 (4)	0.3914 (4)	0.0710 (18)	
H19A	0.3762	0.7083	0.4458	0.107*	
H19B	0.3669	0.8059	0.3945	0.107*	
O20	−0.3399 (9)	0.4351 (5)	0.4577 (4)	0.085 (2)	
H20A	−0.3755	0.4648	0.4079	0.128*	
H20B	−0.2285	0.4429	0.4499	0.128*	
N1	0.3689 (6)	0.9653 (3)	−0.0721 (3)	0.0181 (9)	
N3	0.3478 (6)	0.1321 (3)	0.5096 (3)	0.0227 (9)	
C1	0.2791 (7)	1.0610 (4)	−0.0974 (4)	0.0222 (11)	
H1C	0.2999	1.0985	−0.1608	0.027*	
C2	0.1559 (7)	1.1059 (4)	−0.0317 (4)	0.0197 (10)	
C3	0.1278 (7)	1.0499 (4)	0.0627 (4)	0.0233 (11)	
H3C	0.0490	1.0791	0.1083	0.028*	
C4	0.2181 (7)	0.9499 (4)	0.0887 (3)	0.0218 (11)	
H4C	0.1987	0.9106	0.1515	0.026*	
C5	0.3377 (7)	0.9099 (4)	0.0192 (3)	0.0177 (10)	
C6	0.4413 (7)	0.8016 (4)	0.0383 (3)	0.0186 (10)	
C7	0.0495 (7)	1.2123 (4)	−0.0651 (4)	0.0249 (12)	
C15	0.3497 (8)	0.2265 (4)	0.4463 (4)	0.0238 (11)	
H15A	0.4393	0.2362	0.3904	0.029*	
C16	0.2210 (8)	0.3089 (4)	0.4630 (4)	0.0272 (12)	
C17	0.0938 (9)	0.2969 (5)	0.5474 (4)	0.0359 (15)	
H17A	0.0073	0.3523	0.5596	0.043*	
C18	0.0968 (9)	0.2009 (5)	0.6140 (4)	0.0349 (14)	
H18A	0.0148	0.1912	0.6723	0.042*	
C19	0.2225 (8)	0.1209 (4)	0.5924 (4)	0.0256 (12)	
C20	0.2115 (8)	0.4112 (4)	0.3869 (4)	0.0306 (13)	
C21	0.2323 (8)	0.0112 (4)	0.6572 (4)	0.0254 (12)	
O17	0.3522 (6)	−0.0560 (3)	0.6238 (2)	0.0290 (9)	
O18	0.1287 (6)	−0.0081 (3)	0.7352 (3)	0.0364 (10)	
O13	0.7259 (5)	0.4452 (3)	0.2402 (3)	0.0305 (9)	
C14	0.8891 (8)	0.4189 (4)	0.2009 (4)	0.0257 (12)	
O14	0.9801 (6)	0.4704 (3)	0.1242 (3)	0.0385 (11)	
C9	0.9846 (7)	0.3138 (4)	0.2468 (4)	0.0226 (11)	
C8	1.1424 (8)	0.2750 (4)	0.1928 (4)	0.0255 (12)	
H8A	1.1890	0.3162	0.1329	0.031*	
C10	0.9196 (8)	0.2533 (5)	0.3365 (4)	0.0281 (12)	
H10A	0.8130	0.2777	0.3744	0.034*	
N2	1.2312 (6)	0.1808 (3)	0.2233 (3)	0.0234 (10)	
C11	1.0135 (8)	0.1559 (4)	0.3702 (4)	0.0259 (12)	
H11A	0.9733	0.1152	0.4312	0.031*	
C12	1.1666 (7)	0.1212 (4)	0.3115 (3)	0.0212 (11)	
C13	1.2777 (8)	0.0148 (4)	0.3346 (4)	0.0255 (12)	
O11	1.4056 (5)	−0.0058 (3)	0.2658 (2)	0.0277 (9)	
O12	1.2456 (6)	−0.0441 (3)	0.4144 (3)	0.0398 (11)	

### Atomic displacement parameters (Å^2^)

**Table:**

	*U*^11^	*U*^22^	*U*^33^	*U*^12^	*U*^13^	*U*^23^
Yb1	0.01978 (12)	0.01595 (11)	0.01753 (11)	0.00351 (8)	−0.00040 (8)	−0.00100 (8)
Cu1	0.0237 (3)	0.0165 (3)	0.0195 (3)	0.0044 (3)	0.0007 (2)	−0.0018 (2)
Cu2	0.0298 (5)	0.0168 (4)	0.0217 (4)	0.0030 (4)	−0.0021 (4)	−0.0002 (4)
O1	0.038 (2)	0.026 (2)	0.038 (2)	0.0019 (18)	−0.0167 (19)	−0.0017 (18)
O2	0.046 (3)	0.059 (3)	0.051 (3)	0.016 (2)	−0.021 (2)	−0.033 (2)
O3	0.033 (2)	0.033 (2)	0.033 (2)	0.0115 (19)	0.0083 (18)	0.0084 (18)
O4	0.029 (2)	0.0157 (18)	0.037 (2)	0.0018 (16)	−0.0095 (17)	−0.0048 (16)
O5	0.026 (2)	0.024 (2)	0.031 (2)	0.0091 (17)	0.0019 (16)	0.0034 (17)
O6	0.044 (3)	0.020 (2)	0.031 (2)	0.0068 (18)	−0.0084 (18)	−0.0072 (17)
O7	0.033 (2)	0.0200 (19)	0.0212 (18)	0.0013 (16)	−0.0008 (16)	0.0003 (15)
O8	0.028 (2)	0.0149 (18)	0.0239 (18)	0.0081 (15)	−0.0009 (15)	−0.0035 (15)
O9	0.035 (2)	0.023 (2)	0.035 (2)	0.0063 (18)	−0.0026 (18)	−0.0064 (17)
O10	0.039 (3)	0.029 (2)	0.033 (2)	0.0116 (19)	−0.0024 (19)	0.0016 (18)
O15	0.033 (2)	0.024 (2)	0.037 (2)	0.0072 (18)	0.0082 (19)	0.0093 (18)
O16	0.048 (3)	0.035 (3)	0.053 (3)	0.018 (2)	0.016 (2)	0.011 (2)
O19	0.100 (5)	0.042 (3)	0.059 (3)	0.010 (3)	0.002 (3)	−0.021 (3)
O20	0.080 (5)	0.083 (5)	0.069 (4)	0.009 (4)	−0.001 (3)	−0.005 (3)
N1	0.017 (2)	0.014 (2)	0.022 (2)	0.0012 (17)	−0.0024 (16)	−0.0055 (16)
N3	0.024 (2)	0.020 (2)	0.022 (2)	0.0002 (19)	−0.0041 (18)	−0.0038 (18)
C1	0.023 (3)	0.019 (3)	0.020 (2)	−0.001 (2)	−0.004 (2)	0.000 (2)
C2	0.015 (2)	0.017 (2)	0.029 (3)	0.000 (2)	−0.008 (2)	−0.007 (2)
C3	0.018 (3)	0.026 (3)	0.027 (3)	0.001 (2)	−0.002 (2)	−0.012 (2)
C4	0.021 (3)	0.025 (3)	0.018 (2)	−0.003 (2)	−0.002 (2)	−0.005 (2)
C5	0.016 (2)	0.016 (2)	0.021 (2)	−0.0014 (19)	−0.0055 (19)	−0.0034 (19)
C6	0.018 (3)	0.016 (2)	0.023 (2)	−0.001 (2)	−0.009 (2)	−0.0022 (19)
C7	0.020 (3)	0.021 (3)	0.033 (3)	−0.004 (2)	−0.006 (2)	−0.004 (2)
C15	0.026 (3)	0.022 (3)	0.018 (2)	−0.002 (2)	−0.002 (2)	0.001 (2)
C16	0.034 (3)	0.017 (3)	0.024 (3)	−0.003 (2)	0.000 (2)	−0.002 (2)
C17	0.042 (4)	0.023 (3)	0.030 (3)	0.008 (3)	0.004 (3)	−0.005 (2)
C18	0.039 (4)	0.030 (3)	0.023 (3)	0.001 (3)	0.009 (2)	−0.002 (2)
C19	0.032 (3)	0.021 (3)	0.018 (2)	−0.002 (2)	−0.001 (2)	−0.001 (2)
C20	0.031 (3)	0.021 (3)	0.029 (3)	0.004 (2)	0.001 (2)	0.001 (2)
C21	0.031 (3)	0.020 (3)	0.022 (3)	−0.003 (2)	−0.006 (2)	0.000 (2)
O17	0.037 (2)	0.0181 (19)	0.0229 (19)	0.0017 (17)	−0.0011 (16)	0.0010 (15)
O18	0.045 (3)	0.029 (2)	0.023 (2)	−0.0034 (19)	0.0053 (18)	0.0012 (17)
O13	0.022 (2)	0.034 (2)	0.027 (2)	0.0098 (17)	−0.0028 (16)	−0.0050 (17)
C14	0.026 (3)	0.022 (3)	0.029 (3)	0.003 (2)	−0.005 (2)	−0.009 (2)
O14	0.025 (2)	0.030 (2)	0.039 (2)	0.0053 (18)	0.0023 (18)	0.0106 (18)
C9	0.020 (3)	0.019 (3)	0.030 (3)	0.000 (2)	−0.006 (2)	−0.008 (2)
C8	0.024 (3)	0.024 (3)	0.021 (3)	0.000 (2)	0.002 (2)	−0.002 (2)
C10	0.022 (3)	0.035 (3)	0.024 (3)	0.004 (2)	0.001 (2)	−0.011 (2)
N2	0.023 (2)	0.019 (2)	0.023 (2)	0.0028 (18)	−0.0005 (18)	−0.0028 (18)
C11	0.026 (3)	0.028 (3)	0.021 (3)	−0.003 (2)	−0.002 (2)	−0.006 (2)
C12	0.024 (3)	0.021 (3)	0.017 (2)	−0.001 (2)	−0.004 (2)	−0.001 (2)
C13	0.027 (3)	0.020 (3)	0.025 (3)	0.002 (2)	−0.006 (2)	−0.002 (2)
O11	0.031 (2)	0.0202 (19)	0.0208 (18)	0.0051 (16)	0.0036 (16)	−0.0004 (15)
O12	0.044 (3)	0.035 (2)	0.023 (2)	0.007 (2)	0.0023 (18)	0.0049 (18)

### Geometric parameters (Å, °)

**Table:**

Yb1—O2	2.299 (5)	N1—C5	1.345 (6)
Yb1—O3	2.272 (4)	N3—C15	1.347 (6)
Yb1—O4	2.411 (4)	N3—C19	1.351 (7)
Yb1—O5	2.278 (4)	C1—C2	1.388 (7)
Yb1—O6	2.364 (4)	C1—H1C	0.9300
Yb1—O7	2.447 (3)	C2—C3	1.386 (7)
Yb1—O13	2.364 (4)	C2—C7	1.518 (7)
Yb1—O15	2.281 (4)	C3—C4	1.388 (7)
Cu1—N1	1.975 (4)	C3—H3C	0.9300
Cu1—N2^i^	1.955 (4)	C4—C5	1.384 (7)
Cu1—O1	2.372 (4)	C4—H4C	0.9300
Cu1—O8	1.964 (3)	C5—C6	1.510 (7)
Cu1—O11^i^	1.943 (3)	C15—C16	1.379 (7)
Cu2—N3^ii^	1.967 (4)	C15—H15A	0.9300
Cu2—N3	1.967 (4)	C16—C17	1.376 (7)
Cu2—O17	1.931 (4)	C16—C20	1.515 (7)
Cu2—O17^ii^	1.931 (4)	C17—C18	1.386 (8)
O1—H1A	0.8187	C17—H17A	0.9300
O1—H1B	0.8198	C18—C19	1.365 (8)
O2—H2A	0.8178	C18—H18A	0.9300
O2—H2B	0.8205	C19—C21	1.516 (7)
O3—H3A	0.8205	C21—O18	1.224 (6)
O3—H3B	0.8178	C21—O17	1.285 (6)
O4—H4A	0.8202	O13—C14	1.250 (6)
O4—H4B	0.8200	C14—O14	1.259 (6)
O5—H5A	0.8202	C14—C9	1.516 (7)
O5—H5B	0.8202	C9—C8	1.374 (7)
O6—H6A	0.8201	C9—C10	1.378 (7)
O6—H6B	0.8196	C8—N2	1.331 (7)
O7—C6	1.230 (6)	C8—H8A	0.9300
O8—C6	1.275 (6)	C10—C11	1.388 (8)
O9—C7	1.264 (6)	C10—H10A	0.9300
O10—C7	1.245 (6)	N2—C12	1.356 (6)
O15—C20	1.260 (6)	N2—Cu1^i^	1.955 (4)
O16—C20	1.247 (7)	C11—C12	1.369 (7)
O19—H19A	0.8211	C11—H11A	0.9300
O19—H19B	0.8196	C12—C13	1.514 (7)
O20—H20A	0.8200	C13—O12	1.225 (6)
O20—H20B	0.8196	C13—O11	1.291 (6)
N1—C1	1.338 (6)	O11—Cu1^i^	1.943 (3)
			
O3—Yb1—O5	140.82 (13)	C15—N3—Cu2	129.5 (4)
O3—Yb1—O15	74.65 (14)	C19—N3—Cu2	111.9 (3)
O5—Yb1—O15	143.99 (14)	N1—C1—C2	121.8 (5)
O3—Yb1—O2	87.37 (17)	N1—C1—H1C	119.1
O5—Yb1—O2	93.59 (17)	C2—C1—H1C	119.1
O15—Yb1—O2	94.33 (18)	C3—C2—C1	118.6 (5)
O3—Yb1—O6	120.10 (16)	C3—C2—C7	121.8 (5)
O5—Yb1—O6	77.53 (15)	C1—C2—C7	119.6 (4)
O15—Yb1—O6	76.43 (15)	C2—C3—C4	119.6 (5)
O2—Yb1—O6	146.13 (15)	C2—C3—H3C	120.2
O3—Yb1—O13	140.12 (13)	C4—C3—H3C	120.2
O5—Yb1—O13	76.19 (13)	C5—C4—C3	118.6 (5)
O15—Yb1—O13	72.76 (14)	C5—C4—H4C	120.7
O2—Yb1—O13	72.90 (15)	C3—C4—H4C	120.7
O6—Yb1—O13	73.24 (14)	N1—C5—C4	121.8 (4)
O3—Yb1—O4	77.38 (15)	N1—C5—C6	114.6 (4)
O5—Yb1—O4	79.41 (14)	C4—C5—C6	123.5 (4)
O15—Yb1—O4	111.93 (15)	O7—C6—O8	125.8 (5)
O2—Yb1—O4	144.31 (15)	O7—C6—C5	119.7 (5)
O6—Yb1—O4	66.81 (13)	O8—C6—C5	114.5 (4)
O13—Yb1—O4	136.66 (13)	O10—C7—O9	125.6 (5)
O3—Yb1—O7	68.67 (13)	O10—C7—C2	117.4 (5)
O5—Yb1—O7	74.46 (13)	O9—C7—C2	117.0 (5)
O15—Yb1—O7	141.10 (13)	N3—C15—C16	121.1 (5)
O2—Yb1—O7	71.91 (15)	N3—C15—H15A	119.5
O6—Yb1—O7	133.87 (13)	C16—C15—H15A	119.5
O13—Yb1—O7	131.94 (14)	C17—C16—C15	120.0 (5)
O4—Yb1—O7	72.50 (13)	C17—C16—C20	119.6 (5)
O11^i^—Cu1—N2^i^	83.59 (16)	C15—C16—C20	120.4 (5)
O11^i^—Cu1—O8	168.02 (17)	C16—C17—C18	118.8 (5)
N2^i^—Cu1—O8	94.15 (16)	C16—C17—H17A	120.6
O11^i^—Cu1—N1	97.55 (16)	C18—C17—H17A	120.6
N2^i^—Cu1—N1	170.26 (18)	C19—C18—C17	118.7 (5)
O8—Cu1—N1	82.73 (15)	C19—C18—H18A	120.6
O11^i^—Cu1—O1	105.91 (15)	C17—C18—H18A	120.6
N2^i^—Cu1—O1	95.56 (17)	N3—C19—C18	122.7 (5)
O8—Cu1—O1	86.00 (15)	N3—C19—C21	113.7 (5)
N1—Cu1—O1	93.43 (16)	C18—C19—C21	123.6 (5)
O17—Cu2—O17^ii^	180.0	O16—C20—O15	125.6 (5)
O17—Cu2—N3^ii^	95.81 (16)	O16—C20—C16	117.0 (5)
O17^ii^—Cu2—N3^ii^	84.19 (16)	O15—C20—C16	117.2 (5)
O17—Cu2—N3	84.19 (16)	O18—C21—O17	124.7 (5)
O17^ii^—Cu2—N3	95.81 (16)	O18—C21—C19	119.8 (5)
N3^ii^—Cu2—N3	180.0 (3)	O17—C21—C19	115.5 (4)
Cu1—O1—H1A	100.1	C21—O17—Cu2	114.7 (3)
Cu1—O1—H1B	104.2	C14—O13—Yb1	135.2 (3)
H1A—O1—H1B	106.2	O13—C14—O14	125.9 (5)
Yb1—O2—H2A	118.6	O13—C14—C9	117.7 (5)
Yb1—O2—H2B	132.6	O14—C14—C9	116.4 (5)
H2A—O2—H2B	107.7	C8—C9—C10	118.3 (5)
Yb1—O3—H3A	129.8	C8—C9—C14	117.0 (5)
Yb1—O3—H3B	122.7	C10—C9—C14	124.6 (5)
H3A—O3—H3B	107.3	N2—C8—C9	122.4 (5)
Yb1—O4—H4A	112.3	N2—C8—H8A	118.8
Yb1—O4—H4B	108.2	C9—C8—H8A	118.8
H4A—O4—H4B	105.2	C9—C10—C11	119.9 (5)
Yb1—O5—H5A	122.2	C9—C10—H10A	120.0
Yb1—O5—H5B	125.1	C11—C10—H10A	120.0
H5A—O5—H5B	105.8	C8—N2—C12	119.2 (4)
Yb1—O6—H6A	119.0	C8—N2—Cu1^i^	127.6 (4)
Yb1—O6—H6B	126.0	C12—N2—Cu1^i^	113.1 (3)
H6A—O6—H6B	108.2	C12—C11—C10	118.5 (5)
C6—O7—Yb1	136.8 (3)	C12—C11—H11A	120.8
C6—O8—Cu1	115.5 (3)	C10—C11—H11A	120.8
C20—O15—Yb1	140.2 (4)	N2—C12—C11	121.6 (5)
H19A—O19—H19B	106.7	N2—C12—C13	113.0 (4)
H20A—O20—H20B	108.0	C11—C12—C13	125.3 (5)
C1—N1—C5	119.6 (4)	O12—C13—O11	124.6 (5)
C1—N1—Cu1	128.1 (3)	O12—C13—C12	120.2 (5)
C5—N1—Cu1	112.3 (3)	O11—C13—C12	115.2 (4)
C15—N3—C19	118.6 (5)	C13—O11—Cu1^i^	114.7 (3)
			
O2—Yb1—O7—C6	128.6 (5)	C5—N1—C1—C2	1.0 (8)
O3—Yb1—O7—C6	−137.1 (5)	Cu1—N1—C5—C4	177.6 (4)
O4—Yb1—O7—C6	−54.1 (5)	Cu1—N1—C5—C6	−3.1 (6)
O5—Yb1—O7—C6	29.4 (5)	C1—N1—C5—C4	−1.5 (8)
O6—Yb1—O7—C6	−25.3 (6)	C1—N1—C5—C6	177.7 (5)
O13—Yb1—O7—C6	83.7 (5)	C12—N2—C8—C9	−1.3 (8)
O15—Yb1—O7—C6	−157.6 (5)	Cu1^i^—N2—C8—C9	175.7 (4)
O2—Yb1—O13—C14	−109.4 (5)	C8—N2—C12—C11	−0.6 (8)
O3—Yb1—O13—C14	−173.1 (5)	C8—N2—C12—C13	177.9 (5)
O4—Yb1—O13—C14	46.4 (6)	Cu1^i^—N2—C12—C11	−178.0 (4)
O5—Yb1—O13—C14	−11.1 (5)	Cu1^i^—N2—C12—C13	0.5 (6)
O6—Yb1—O13—C14	69.8 (5)	Cu2—N3—C15—C16	174.8 (4)
O7—Yb1—O13—C14	−64.9 (6)	C19—N3—C15—C16	−3.2 (8)
O15—Yb1—O13—C14	150.4 (6)	Cu2—N3—C19—C18	−177.7 (5)
O2—Yb1—O15—C20	80.6 (6)	Cu2—N3—C19—C21	1.0 (6)
O3—Yb1—O15—C20	−5.5 (6)	C15—N3—C19—C18	0.7 (9)
O4—Yb1—O15—C20	−74.7 (6)	C15—N3—C19—C21	179.4 (5)
O5—Yb1—O15—C20	−177.2 (5)	N1—C1—C2—C3	0.8 (8)
O6—Yb1—O15—C20	−132.4 (6)	N1—C1—C2—C7	−176.6 (5)
O7—Yb1—O15—C20	14.3 (7)	C1—C2—C3—C4	−2.0 (8)
O13—Yb1—O15—C20	151.2 (6)	C7—C2—C3—C4	175.3 (5)
O1—Cu1—O8—C6	−90.2 (4)	C1—C2—C7—O9	173.9 (5)
N1—Cu1—O8—C6	3.8 (4)	C1—C2—C7—O10	−4.5 (8)
N2^i^—Cu1—O8—C6	174.6 (4)	C3—C2—C7—O9	−3.4 (8)
O1—Cu1—N1—C1	−95.5 (5)	C3—C2—C7—O10	178.2 (5)
O1—Cu1—N1—C5	85.5 (4)	C2—C3—C4—C5	1.5 (8)
O8—Cu1—N1—C1	179.0 (5)	C3—C4—C5—N1	0.3 (8)
O8—Cu1—N1—C5	−0.1 (4)	C3—C4—C5—C6	−178.9 (5)
O11^i^—Cu1—N1—C1	11.1 (5)	N1—C5—C6—O7	−173.5 (5)
O11^i^—Cu1—N1—C5	−168.0 (4)	N1—C5—C6—O8	6.4 (7)
O1—Cu1—O11^i^—C13^i^	−88.6 (4)	C4—C5—C6—O7	5.7 (8)
N1—Cu1—O11^i^—C13^i^	175.6 (4)	C4—C5—C6—O8	−174.4 (5)
O1—Cu1—N2^i^—C8^i^	−79.9 (5)	N2—C8—C9—C10	1.5 (9)
O1—Cu1—N2^i^—C12^i^	103.0 (4)	N2—C8—C9—C14	−175.7 (5)
O8—Cu1—N2^i^—C8^i^	6.6 (5)	C8—C9—C10—C11	0.1 (9)
O8—Cu1—N2^i^—C12^i^	−170.6 (4)	C14—C9—C10—C11	177.1 (6)
N3—Cu2—O17—C21	−1.5 (4)	C8—C9—C14—O13	165.0 (5)
O12^iii^—Cu2—O17—C21	−92.1 (4)	C8—C9—C14—O14	−12.6 (8)
N3^ii^—Cu2—O17—C21	178.5 (4)	C10—C9—C14—O13	−12.0 (9)
O12^iv^—Cu2—O17—C21	88.0 (4)	C10—C9—C14—O14	170.4 (6)
O17—Cu2—N3—C15	−178.0 (5)	C9—C10—C11—C12	−1.9 (9)
O17—Cu2—N3—C19	0.2 (4)	C10—C11—C12—N2	2.2 (8)
O12^iii^—Cu2—N3—C15	−86.7 (5)	C10—C11—C12—C13	−176.1 (5)
O12^iii^—Cu2—N3—C19	91.5 (4)	N2—C12—C13—O11	−4.9 (7)
O17^ii^—Cu2—N3—C15	2.0 (5)	N2—C12—C13—O12	173.9 (5)
O17^ii^—Cu2—N3—C19	−179.8 (4)	C11—C12—C13—O11	173.5 (5)
O12^iv^—Cu2—N3—C15	93.3 (5)	C11—C12—C13—O12	−7.7 (9)
O12^iv^—Cu2—N3—C19	−88.6 (4)	N3—C15—C16—C17	3.2 (9)
Yb1—O7—C6—O8	−17.4 (9)	N3—C15—C16—C20	−173.5 (5)
Yb1—O7—C6—C5	162.5 (4)	C15—C16—C17—C18	−0.7 (9)
Cu1—O8—C6—O7	173.5 (4)	C20—C16—C17—C18	176.1 (6)
Cu1—O8—C6—C5	−6.4 (6)	C15—C16—C20—O15	−16.8 (8)
Cu1^i^—O11—C13—O12	−171.8 (5)	C15—C16—C20—O16	159.0 (6)
Cu1^i^—O11—C13—C12	7.0 (6)	C17—C16—C20—O15	166.5 (6)
Cu2^v^—O12—C13—O11	85.5 (6)	C17—C16—C20—O16	−17.7 (9)
Cu2^v^—O12—C13—C12	−93.1 (5)	C16—C17—C18—C19	−1.8 (10)
Yb1—O13—C14—O14	25.3 (9)	C17—C18—C19—N3	1.8 (10)
Yb1—O13—C14—C9	−152.0 (4)	C17—C18—C19—C21	−176.7 (6)
Yb1—O15—C20—O16	11.1 (10)	N3—C19—C21—O17	−2.2 (8)
Yb1—O15—C20—C16	−173.6 (4)	N3—C19—C21—O18	178.3 (5)
Cu2—O17—C21—O18	−178.2 (5)	C18—C19—C21—O17	176.4 (6)
Cu2—O17—C21—C19	2.4 (6)	C18—C19—C21—O18	−3.1 (9)
Cu1—N1—C1—C2	−178.0 (4)		

Symmetry codes: (i) −*x*+2, −*y*+1, −*z*; (ii) −*x*+1, −*y*, −*z*+1; (iii) *x*−1, *y*, *z*; (iv) −*x*+2, −*y*, −*z*+1; (v) *x*+1, *y*, *z*.

### Hydrogen-bond geometry (Å, °)

**Table:**

*D*—H···*A*	*D*—H	H···*A*	*D*···*A*	*D*—H···*A*
O1—H1A···O17^vi^	0.82	2.50	3.072 (5)	128
O1—H1A···O18^vi^	0.82	2.06	2.865 (6)	166
O1—H1B···O9^vii^	0.82	2.02	2.840 (6)	173
O2—H2A···O10^viii^	0.82	2.48	2.988 (7)	121
O2—H2B···O19	0.82	1.83	2.644 (8)	173
O3—H3A···O16	0.82	1.91	2.572 (6)	137
O3—H3B···O10^vii^	0.82	1.91	2.695 (6)	159
O4—H4A···O9^vii^	0.82	1.87	2.670 (6)	165
O4—H4B···O14^iii^	0.82	1.98	2.774 (6)	163
O5—H5A···O14	0.82	1.85	2.586 (6)	148
O5—H5B···O8	0.82	2.06	2.717 (6)	137
O6—H6A···O1^ix^	0.82	2.09	2.826 (6)	149
O6—H6B···O4^ix^	0.82	2.15	2.936 (6)	160
O19—H19A···O20^x^	0.82	2.06	2.816 (8)	152
O19—H19B···O12^xi^	0.82	2.15	2.965 (7)	171
O20—H20A···O13^iii^	0.82	2.55	3.174 (7)	134
O20—H20B···O16	0.82	2.15	2.957 (9)	166
C3—H3C···O18^x^	0.93	2.42	3.134 (7)	134
C11—H11A···O12^iv^	0.93	2.52	3.409 (7)	159

Symmetry codes: (vi) *x*, *y*+1, *z*−1; (vii) −*x*, −*y*+2, −*z*; (viii) −*x*+1, −*y*+2, −*z*; (iii) *x*−1, *y*, *z*; (ix) −*x*+1, −*y*+1, −*z*; (x) −*x*, −*y*+1, −*z*+1; (xi) *x*−1, *y*+1, *z*; (iv) −*x*+2, −*y*, −*z*+1.
